# Codex (Cognitive Disorders Examination) Decision Tree Modified for the Detection of Dementia and MCI

**DOI:** 10.3390/diagnostics9020058

**Published:** 2019-06-01

**Authors:** Besa Ziso, Andrew J. Larner

**Affiliations:** Cognitive Function Clinic, Walton Centre for Neurology and Neurosurgery, Liverpool L9 7LJ, UK; besa.Ziso@thewaltoncentre.nhs.uk

**Keywords:** Codex, decision tree, dementia, Free-Cog, MoCA, mild cognitive impairment, sensitivity and specificity

## Abstract

Many cognitive screening instruments are available to assess patients with cognitive symptoms in whom a diagnosis of dementia or mild cognitive impairment is being considered. Most are quantitative scales with specified cut-off values. In contrast, the cognitive disorders examination or Codex is a two-step decision tree which incorporates components from the Mini-Mental State Examination (MMSE) (three word recall, spatial orientation) along with a simplified clock drawing test to produce categorical outcomes defining the probability of dementia diagnosis and, by implication, directing clinician response (reassurance, monitoring, further investigation, immediate treatment). Codex has been shown to have high sensitivity and specificity for dementia diagnosis but is less sensitive for the diagnosis of mild cognitive impairment (MCI). We examined minor modifications to the Codex decision tree to try to improve its sensitivity for the diagnosis of MCI, based on data extracted from studies of two other cognitive screening instruments, the Montreal Cognitive Assessment and Free-Cog, which are more stringent than MMSE in their tests of delayed recall. Neither modification proved of diagnostic value for mild cognitive impairment. Possible explanations for this failure are considered.

## 1. Introduction

**Figure 1:**
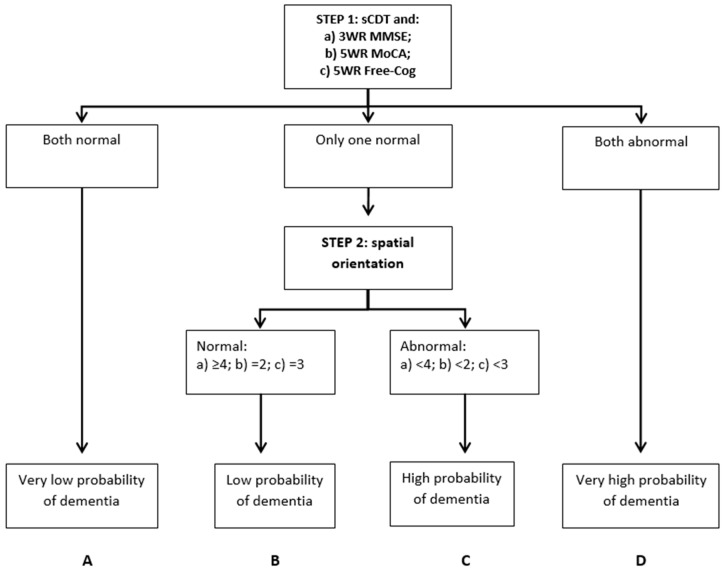
Codex decision tree: (**a**) Original Codex categories (from Belmin et al. 2007 [1]); (**b**) modified Codex categories from MoCA; (**c**) modified Codex categories from Free-Cog.

The cognitive disorders examination or Codex for the detection of dementia described by Belmin et al. [1,2] is a two-step decision tree for diagnostic prediction, developed by identifying independent variables related to dementia using a multivariable logistic model. Binary recursive partitioning incorporated the three-word recall and spatial orientation components from the Mini-Mental State Examination (MMSE) [3] along with a simplified clock drawing test (sCDT, scored 1 or 0, respectively, normal and abnormal) to produce four terminal nodes, the endpoint values having different probabilities of dementia diagnosis (categories A–D, respectively, with very low, low, high, and very high probability of dementia; Figure 1a). Codex takes around three minutes to perform. In the index study, Codex had both high sensitivity and specificity for the diagnosis of dementia (0.92 and 0.85, respectively) [1].

An independent pragmatic test accuracy study of Codex, undertaken by Ziso and Larner in a dedicated cognitive disorders clinic in a secondary care setting [4,5,6], confirmed good sensitivity and specificity for dementia diagnosis (0.84 and 0.82, respectively), as did a proof of concept study of a Greek translation of Codex based in a primary care setting (sensitivity 0.94, specificity 0.89) [7].

Codex has also found other applications, for example, in predicting postoperative delirium in patients undergoing femoral fracture repair [8] and in monitoring cognitive impairment before and after cochlear implantation surgery [9]. Codex was included as part of the protocol of the EVATEM study for the detection of cognitive disorders amongst community-dwelling elderly people with memory complaints [10].

Despite the excellent metrics for dementia detection, Codex performance in detecting mild cognitive impairment (MCI), often a prodrome to dementia, is less certain. Ziso and Larner found that for a diagnosis of any cognitive impairment, dementia or MCI, Codex sensitivity was lower (0.68) whilst specificity was improved (0.91) compared to dementia detection [5,6]. In the EVATEM study (see Table 4 in [11]), Codex was found to have low sensitivity (0.32) but high specificity (0.85) for the detection of cognitive impairment (MCI and dementia, of which MCI patients made up a large majority, 176/182). These data suggest that Codex as originally formulated may be insufficiently sensitive for the detection of MCI.

We reasoned that minor modifications to the Codex decision tree might improve its screening utility for MCI. Specifically, since most MCI, whether of single or multiple domain type, includes an amnestic component, the use of a more stringent delayed recall paradigm might result in an instrument more sensitive to lesser degrees of cognitive impairment. Both the Montreal Cognitive Assessment (MoCA) [12] and the recently described Free-Cog (Professor Alistair Burns, Manchester, UK, personal communication, 2017) have a delayed recall test of five words rather than three words as in the MMSE [3]. Deriving a modified Codex from these instruments might therefore increase sensitivity for MCI, as shown in head to head studies of MoCA and MMSE [12,13], even allowing for the fewer spatial orientation components in these tests (MoCA 2, Free-Cog 3, versus MMSE 5).

We analysed data from pragmatic test accuracy studies of MoCA [14] and Free-Cog [15] and also reanalyzed data from a previous Codex test accuracy study [5,6] to examine whether a modified Codex might improve diagnostic utility for MCI.

## 2. Materials and Methods

Data from consecutive patient cohorts, referred to a dedicated cognitive function clinic based in secondary care and who were administered either MoCA (June 2015–May 2016 inclusive) [14] or Free-Cog (November 2017–October 2018 inclusive) [15], were analysed. Data from a previous consecutive patient cohort, referred to the same clinic and who were administered MMSE and sCDT (February–November 2012 inclusive) [5,6], were reanalysed. Standard diagnostic criteria for dementia (DSM-IV) and MCI [16] were used in these studies. Criterion diagnosis was by the judgment of an experienced clinician based on diagnostic criteria.

In the modified Codex decision tree derived from MoCA (Figure 1b), there were five delayed recall components but only two spatial orientation components [12], whereas in modified Codex derived from Free-Cog (Figure 1c), there were five delayed recall components but only three spatial orientation components. Both MoCA and Free-Cog incorporate clock drawing tests, unlike the MMSE, the scoring for which was simplified to 1 or 0 depending on whether or not all elements were completed correctly, as per the original Codex [1].

Categorical data were derived from the decision trees with differing probabilities of diagnosis (A = very low, B = low, C = high, D = very high), with categories C and D taken to be indicators of cognitive impairment [1]. Codex categories were not used in reference diagnosis to avoid review bias.

Dependent on the date of the study, either STARD or STARDdem guidelines for reporting diagnostic test accuracy studies in dementia [17,18] were observed. Standard summary measures of discrimination were calculated: sensitivity and specificity, false positive and false negative rates, Youden index (Y), positive and negative predictive values (PPV, NPV), predictive summary index (PSI), accuracy, net reclassification improvement (NRI), positive and negative likelihood ratios (LR+, LR−), diagnostic odds ratio (DOR), and clinical utility indexes (CUI+, CUI−). The recently described “likelihood to be diagnosed or misdiagnosed” (LDM) metric, the ratio of number needed to misdiagnose (NNM = 1/(1 – Accuracy)) to either number needed to diagnose (NND = 1/Y) or number needed to predict (NNP = 1/PSI), was also calculated; desirably, tests have LDM >1 [19].

## 3. Results

**Figure 2:**
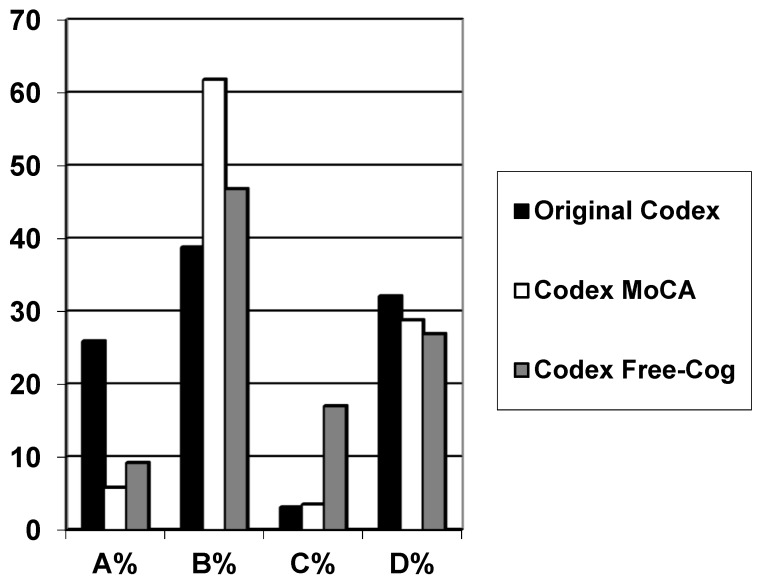
Codex categories versus patient diagnosis for each formulation of the decision tree; A = very low probability of dementia; B = low probability of dementia; C = high probability of dementia; D = very high probability of dementia (see Figure 1).

Baseline demographic data from the studies examining original Codex and modified Codex derived from MoCA or Free-Cog are shown in Table 1, along with the distribution of observed Codex categories versus diagnosis for each formulation of the decision tree (Figure 2).

**Figure 3:**
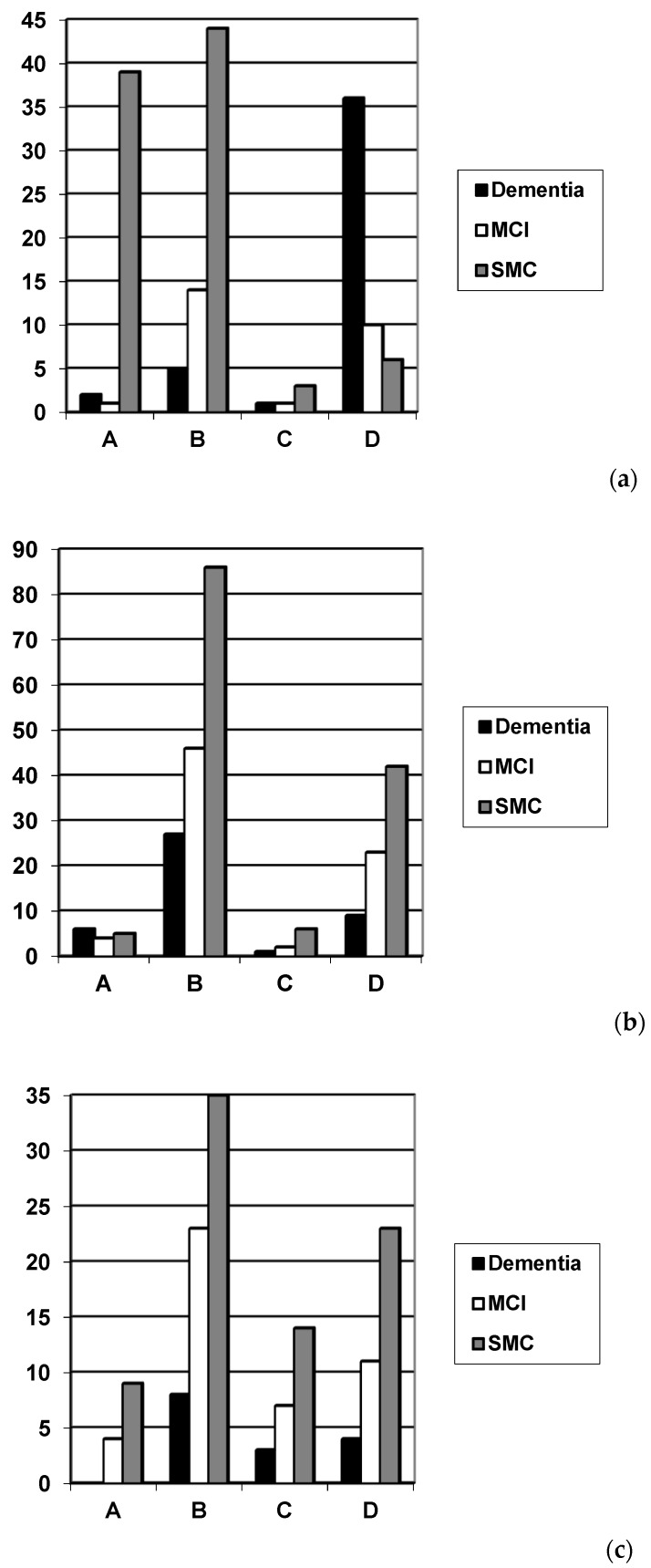
Diagnosis (dementia/MCI/SMC) plotted against: (**a**) Original Codex category (adapted from Ziso and Larner 2013 [5]); (**b**) Modified Codex category from MoCA; (**c**) Modified Codex category from Free-Cog; A = very low probability of dementia; B = low probability of dementia; C = high probability of dementia; D = very high probability of dementia (see Figure 1).

Measures of discrimination showed original Codex achieved very good sensitivity and specificity for the diagnosis of dementia versus no dementia, very good sensitivity for the diagnosis of dementia versus MCI, and very good specificity for the diagnosis of MCI versus no dementia (Table 2 and Figure 3a).

Measures of discrimination showed modified Codex derived from either MoCA (Table 3 and Figure 3b) or Free-Cog (Table 4 and Figure 3c) had lower sensitivity and specificity for both dementia and MCI diagnosis. For all parameters examined, original Codex performed better than either modified Codex.

## 4. Discussion

The Codex decision tree proved easy to use, in both its original and modified forms. In particular, the ease of data extraction for modified Codex from both MoCA and Free-Cog required no extra sCDT step as required for original Codex derived from MMSE.

The performance of the original Codex for MCI diagnosis was very similar to that observed in the EVATEM study, namely, excellent specificity (0.90 vs. 0.85) and good NPV (0.85 vs 0.74) but poor sensitivity (0.42 vs 0.32) and modest PPV (0.55 vs 0.48). Original Codex therefore appears to be a test which is poor for ruling in a diagnosis of MCI.

The hope that minor modifications of the original Codex decision tree would afford better performance for MCI detection was not realized, with all parameters worse than for original Codex. Possible reasons for this failure might relate to the case mix in the various studies examined. There was an inversion in the frequency of dementia and MCI in the studies examining modified Codex compared to original Codex (see Table 1), reflecting changes in referral practice to the clinic. This changed the pretest odds of diagnosis in the different cohorts, specifically, in the latter cohorts examined with the modified forms of Codex, the pretest odds of MCI were higher than in the cohort administered original Codex. The typical shortcomings of clinic-based studies, such as the use of cross-sectional clinical diagnosis without delayed verification, are unlikely to explain the findings since this methodology was consistent between the study cohorts.

Many patients diagnosed clinically with subjective memory complaint were classified in category D (= very high probability of dementia) in the modified Codex decision trees (Figure 3b,c), hence false positives, suggesting inadequate test specificity. This might be anticipated if the modified Codex is, as hoped, more sensitive to cognitive impairment as a consequence of the changed (more stringent) delayed recall testing. However, some patients diagnosed clinically with dementia were nevertheless classified in category B (= low probability of dementia) in the modified Codex decision trees, hence false negatives, suggesting inadequate sensitivity. This might be a consequence of the changed (less stringent) spatial awareness testing.

Limitations of the study include the use of clinical diagnostic criteria for dementia and MCI, and the cross-sectional design which risks some miscategorisation of cases. The use of clinico-biological criteria incorporating imaging and CSF biomarkers [20] (not available to us) and longitudinal follow-up for the delayed verification of diagnosis might circumvent these problems. Moreover, “dementia” and “MCI” are broad categories which encompass a variety of neuropathological entities. Whilst Codex analysis by specific diagnosis might be desirable, this was not feasible with the small numbers of dementia and MCI cases (see Table 1). Furthermore, the goal of Codex, as for other cognitive screening instruments, is to identify those patients in whom additional testing is indicated to permit more fine-grained diagnostic classification. Screening tests can only screen for certain types of cognitive impairment related to dementia.

More broadly, this study poses questions about the value of decision trees and categorical data derived therefrom as opposed to the use of standard cognitive screening instruments (CSIs) generating quantitative data. Advantages of decision trees include the way in which they can facilitate medical decision making. The combination of choices from which the categories in a decision tree are derived may be taken to imply distinct management policies, hence in Codex, the categories A and B may result in patient reassurance, whilst categories C and D mandate further investigation, if necessary by onward referral to specialized services. However, medical decision-making policies may be less clear when using numerical cut-offs, although some CSIs, such as DemTect, further categorise cut-off scores in terms of suggested management policies [21]. In the future, the use of computerized techniques based on machine learning may provide better analyses than a decision tree [22,23].

The outcomes with modified Codex were less good than for the base tests (MoCA, Free-Cog), which generate quantitative data and from which Codex was extracted. The additional components in these CSIs may therefore add something which permits a more accurate diagnosis of MCI; data suggesting that CSI length (number of test items) correlates positively with measures of diagnostic accuracy have been presented [24]. Interestingly, in the EVATEM study, the best performance was found when all three tests examined (Codex, five-word test, and verbal fluency) were combined [11]. Whilst test brevity and the easy categorization of results is desirable in time-limited settings such as primary care, a different dispensation applies in dedicated cognitive disorders clinics based in secondary or tertiary care settings.

These data suggest that Codex, in either its original or modified form, is not sensitive for the diagnosis of MCI. Simple modifications of the decision tree which were anticipated to improve MCI detection did not produce the desired outcome, suggesting that tree impurity was not reduced, and that the modified tree was too shallow to identify MCI cases reliably. Hence, other instruments such as MoCA [12], MACE [14,25], and the Quick Mild Cognitive Impairment (Qmci) screen [26], some specifically designed for MCI identification, should be recommended for MCI diagnosis in preference to Codex.

## 5. Conclusions

Minor modifications to the Codex decision tree failed to improve diagnostic value, in particular sensitivity, for mild cognitive impairment.

## Figures and Tables

**Table 1:** Study demographics and base category data.

Codex	N	Gender F:M (% Female)	Age Range (Median)	Diagnosis Dementia/MCI/SMC (%)	Codex Category			
					A (%)	B (%)	C (%)	D (%)
Original	162	79:83 (49)	20–89 (61)	44/26/92 (27/16/57)	42 (25.9)	63 (38.8)	5 (3.1)	52 (32.1)
Modified from MoCA	257	116:141 (45)	22–89 (59)	43/75/139 (17/29/54)	15 (5.8)	159 (61.8)	9 (3.5)	74 (28.8)
Modified from Free-Cog	141	61:80 (43)	28–88 (62)	15/45/81 (11/32/57)	13 (9.2)	66 (46.8)	24 (17.0)	38 (26.9)

Abbreviations: MCI: mild cognitive impairment; SMC: subjective memory complaint; MoCA: Montreal Cognitive Assessment.

**Table 2:** Measures of discrimination for original Codex for the diagnosis of dementia and of MCI (with 95% confidence intervals).

	Diagnosis of Dementia vs. No Dementia (MCI + SMC)	Diagnosis of Dementia vs. MCI	Diagnosis of MCI vs. No Cognitive Impairment (SMC)
N	162 (44 vs. 118)	70 (44 vs. 26)	118 (26 vs. 92)
Prevalence (P = pre-test probability)	Dementia 0.27	Dementia 0.63	MCI 0.22
Pre-test odds (= P/1 − P)	Dementia 0.37	Dementia 1.69	MCI 0.28
Sensitivity (Se)	0.84 (0.73–0.95)	0.84 (0.73–0.95)	0.42 (0.23–0.61)
Specificity (Sp)	0.83 (0.76–0.90)	0.58 (0.39–0.77)	0.90 (0.84–0.96)
Y	0.67	0.42	0.32
PPV (= post-test probability)	0.65 (0.53–0.77)	0.77 (0.65–0.89)	0.55 (0.33–0.77)
NPV	0.93 (0.89–0.98)	0.68 (0.49–0.88)	0.85 (0.78–0.92)
PSI	0.58	0.45	0.40
Accuracy (Acc)	0.83 (0.78–0.89)	0.74 (0.64–0.85)	0.80 (0.72–0.87)
Net Reclassification Improvement (NRI = Acc − P)	0.56	0.11	0.58
LDM (= NNM/NND, NNM/NNP)	4.02, 3.48	1.63, 1.75	1.57, 1.97
LR+	4.96 (3.29–7.49) = moderate	1.99 (1.25–3.17) = slight	4.32 (2.01–9.30) = moderate
LR−	0.19 (0.13–0.29) = large	0.28 (0.17–0.44) = moderate	0.64 (0.30–1.38) = slight
DOR	25.9 (17.2–39.1)	7.21 (4.52–11.5)	6.76 (3.14–14.5)
Post-test odds (= pre-test odds × LR+)	Dementia 1.85	Dementia 3.36	MCI 1.21
CUI+ (= Se × PPV)	0.55 = adequate	0.65 = good	0.23 = very poor
CUI− (= Sp × NPV)	0.78 = good	0.39 = poor	0.76 = good

Abbreviations: MCI: mild cognitive impairment; SMC: subjective memory complaint; Y: Youden index; PPV: positive predictive value; NPV: negative predictive value; PSI: Predictive Summary Index; LDM: likelihood to diagnose or misdiagnose; NNM: number needed to misdiagnose; NND: number needed to diagnose; NNP: number needed to predict; LR+: positive likelihood ratio; LR−: negative likelihood ratio; DOR: diagnostic odds ratio; CUI+: positive clinical utility index; CUI−: negative clinical utility index.

**Table 3:** Measures of discrimination for modified Codex derived from MoCA for the diagnosis of dementia and of MCI (with 95% confidence intervals).

	Diagnosis of Dementia vs. No Dementia (MCI + SMC)	Diagnosis of Dementia vs. MCI	Diagnosis of MCI vs. No Cognitive Impairment (SMC)
N	257 (43 vs. 214)	118 (43 vs. 75)	214 (75 vs. 139)
Prevalence (P = pre-test probability)	Dementia 0.17	Dementia 0.36	MCI 0.35
Pre-test odds (= P/1 − P)	Dementia 0.20	Dementia 0.57	MCI 0.54
Sensitivity (Se)	0.23 (0.11–0.36)	0.23 (0.11–0.36)	0.33 (0.23–0.44)
Specificity (Sp)	0.66 (0.60–0.72)	0.67 (0.56–0.77)	0.65 (0.58–0.73)
Y	−0.11	−0.10	−0.02
PPV (= post-test probability)	0.12 (0.05–0.19)	0.29 (0.14–0.44)	0.34 (0.23–0.45)
NPV	0.81 (0.75–0.87)	0.60 (0.50–0.71)	0.65 (0.57–0.72)
PSI	−0.07	−0.11	−0.01
Accuracy (Acc)	0.59 (0.53–0.65)	0.51 (0.42–0.60)	0.54 (0.48–0.61)
Net Reclassification Improvement (NRI = Acc − P)	0.42	0.15	0.19
LDM (= NNM/NND, NNM/NNP)	−0.27, −0.17	−0.20, −0.22	−0.04, −0.02
LR+	0.68 (0.38–1.21) = slight	0.70 (0.37–1.31) = slight	0.97 (0.65–1.43) = slight
LR−	1.16 (0.66–2.07) = slight	1.15 (0.61–2.16) = slight	1.02 (0.69–1.51) = slight
DOR	0.59 (0.33–1.04)	0.61 (0.32–1.14)	0.95 (0.64–1.40)
Post-test odds (= pre-test odds × LR+)	Dementia 0.14	Dementia 0.40	MCI 0.52
CUI+ (= Se × PPV)	0.03 = very poor	0.07 = very poor	0.11 = very poor
CUI− (= Sp × NPV)	0.53 = adequate	0.40 = poor	0.42 = poor

Abbreviations: MCI: mild cognitive impairment; SMC: subjective memory complaint; Y: Youden index; PPV: positive predictive value; NPV: negative predictive value; PSI: Predictive Summary Index; LDM: likelihood to diagnose or misdiagnose; NNM: number needed to misdiagnose; NND: number needed to diagnose; NNP: number needed to predict; LR+: positive likelihood ratio; LR−: negative likelihood ratio; DOR: diagnostic odds ratio; CUI+: positive clinical utility index; CUI−: negative clinical utility index.

**Table 4:** Measures of discrimination for modified Codex derived from Free-Cog for the diagnosis of dementia and of MCI (with 95% confidence intervals).

	Diagnosis of Dementia vs. No Dementia (MCI + SMC)	Diagnosis of Dementia vs. MCI	Diagnosis of MCI vs. No Cognitive Impairment (SMC)
N	141 (15 vs. 126)	60 (15 vs. 45)	126 (45 vs. 81)
Prevalence (P = pre-test probability)	Dementia 0.11	Dementia 0.25	MCI 0.36
Pre-test odds (= P/1 − P)	Dementia 0.12	Dementia 0.33	MCI 0.56
Sensitivity (Se)	0.47 (0.21–0.72)	0.47 (0.21–0.72)	0.40 (0.26–0.54)
Specificity (Sp)	0.56 (0.48–0.65)	0.60 (0.47–0.74)	0.54 (0.43–0.65)
Y	0.03	0.07	−0.06
PPV (= post-test probability)	0.11 (0.03–0.19)	0.28 (0.10–0.46)	0.33 (0.20–0.45)
NPV	0.90 (0.83–0.97)	0.77 (0.63–0.91)	0.62 (0.51–0.73)
PSI	0.01	0.05	−0.05
Accuracy (Acc)	0.55 (0.47–0.64)	0.57 (0.44–0.69)	0.49 (0.40–0.58)
Net Reclassification Improvement (NRI = Acc − P)	0.44	0.32	0.13
LDM (= NNM/NND, NNM/NNP)	0.07, 0.02	0.63, 0.12	−0.12, −0.10
LR+	1.07 (0.60–1.91) = slight	1.17 (0.61–2.23) = slight	0.88 (0.57–1.35) = slight
LR−	0.95 (0.53–1.68) = slight	0.89 (0.46–1.70) = slight	1.10 (0.72–1.70) = slight
DOR	1.13 (0.63–2.01)	1.31 (0.69–2.51)	0.79 (0.52–1.22)
Post-test odds (= pre-test odds × LR+)	Dementia 0.13	Dementia 0.38	MCI 0.49
CUI+ (= Se × PPV)	0.05 = very poor	0.13 = very poor	0.13 = very poor
CUI− (= Sp × NPV)	0.51 = adequate	0.46 = poor	0.34 = very poor

Abbreviations: MCI: mild cognitive impairment; SMC: subjective memory complaint; Y: Youden index; PPV: positive predictive value; NPV: negative predictive value; PSI: Predictive Summary Index; LDM: likelihood to diagnose or misdiagnose; NNM: number needed to misdiagnose; NND: number needed to diagnose; NNP: number needed to predict; LR+: positive likelihood ratio; LR−: negative likelihood ratio; DOR: diagnostic odds ratio; CUI+: positive clinical utility index; CUI−: negative clinical utility index.
